# Bibliometric Analysis of the 100 Most-Cited Articles on Endodontic Surgery

**DOI:** 10.7759/cureus.85946

**Published:** 2025-06-13

**Authors:** Swapna Munaga, Ali Alaqla, Abdulmohsen Alfadley, Khalid Alfouzan, Ikram UI Haq, Rajkiran Chitumalla

**Affiliations:** 1 Department of Restorative and Prosthetic Dental Sciences, College of Dentistry, King Saud Bin Abdulaziz University for Health Sciences, King Abdullah International Medical Research Center, Ministry of National Guard-Health Affairs, Riyadh, SAU; 2 College of Dentistry, King Saud Bin Abdulaziz University for Health Sciences, Riyadh, SAU

**Keywords:** bibliometrics, citation analysis, endodontic surgery, periapical lesion, root end filling material

## Abstract

Endodontic surgery (ES) includes numerous methods for treating teeth with a history of failed root canal treatment. This 49-year bibliometric analysis aimed to identify the 100 most-cited papers on ES published during 1976-2024 and examine their bibliometric properties. The top 100 cited publications from the Web of Science (WoS) database on ES were used as the dataset for the bibliometric research. Information regarding the publication year, single or multiple authorship, clinical or non-clinical research, mode of accessibility, level of evidence (LOE), geographic location, institution, authors, and keywords was extracted for each article. The VOSviewer (v.1.6.10; https://www.vosviewer.com) was used for co-author, co-citation, and keyword analysis. SPSS version 27 was used for data analysis. Most papers (n = 88) were original research articles, in contrast to review articles (n = 12). Articles authored by multiple authors gathered higher citations. LOE II exhibited the greatest number of publications (25%). Cohort (22%) and cross-sectional studies (14%) were the most common research designs. Over one-third of publications came from the United States, with the University of Pennsylvania being the top producer. The Journal of Endodontics published more than half of the articles. Euiseong Kim authored the majority of the papers, followed by Syngcuk Kim. The most influential keywords were healing, MTA (mineral trioxide aggregate), and periapical surgery. This research reveals a vibrant and growing academic environment worldwide, with a notable uptick in the past 20 years. The results of this study show how important international collaboration is to the advancement of ES and allow readers to identify the prolific authors, countries, organizations, and journals working in this area. Among all fields of study, outcomes of ES have the most-cited articles, followed by root-end filling materials. Clinical studies were more prevalent, with cohort studies being the most preferred study design.

## Introduction and background

As an accurate, scientifically grounded supplement to non-surgical root canal treatment, endodontic surgery (ES) has emerged as a viable option [1]. When conventional root canal therapy fails in clinical conditions, non-surgical retreatment is usually the preferred next step. However, complex root canal systems or previous procedural accidents may limit the success of non-surgical retreatment. In these scenarios, the best option for saving the tooth is periradicular surgery [2].

ES includes numerous methods for treating teeth with a history of failed root canal treatment, including retrograde surgery, surgical repair of perforation, resections of crown and root, and intentional reimplantation. Microsurgical endodontics is an advancement over traditional apicoectomy procedures by the integration of high magnification, ultrasonic root-end preparation, and root-end filling with biocompatible materials [1].

Bibliometric analysis is an effective technique for delineating the intellectual framework of a specific academic discipline. This is a retrospective approach employed to assess and analyze the scientific influence of research within a certain domain [3]. Academic institutions, dental researchers, professional bodies, and funding agencies generally perform bibliometric assessments to develop policies and implement necessary actions [4].

Based on the citation metrics, the bibliometric analysis generates a dataset of the most-cited articles in a certain field of knowledge, allowing researchers to better understand research trends [5]. The publications that are frequently cited by peer researchers in their scholarly work are considered to be among the most cited [6]. Citations have the potential to be used as indicators of a publication’s influence in the context of the constantly growing body of scientific literature [7].

The level of evidence (LOE) rating system is used to assess the methodological quality of the research [8]. Higher levels of evidence generally represent a more rigorous methodology that yields reliable and reproducible results [9]. The Oxford Center for Evidence-based Medicine LOE is a prominent ranking system that has been widely used and adapted by numerous journals and researchers [10].

Numerous studies have examined trends in endodontic research through bibliometric analyses, assessing the most-cited articles in regenerative endodontics, management of fractured instruments, and scientometrics of endodontic journals [11-13]. Despite the large amount of scientific material published on surgical endodontics, there have been no bibliometric studies in this field of dentistry. Hence, this study aimed to identify the 100 most-cited articles on ES and analyze the productive years, type of research, LOE, keywords, top cited authors, institutions, geographic location and field of study.

## Review

Methodology

Search Strategy

The study utilized the quantitative bibliometric approach to the data set obtained from the Expanded Science Citation Index database retrieved on October 27, 2024, via the Web of Science (WoS) Core Collection. The phrases utilized for the search strategy were “endodontic surgery” OR “surgical endodontics” OR “periradicular surgery” OR “endodontic microsurgery” OR “microsurgical endodontics” OR “endodontic retrograde surgery” OR “periapical surgery” OR “apicoectomy” OR “retrofilling” OR “micro endodontic surgery” OR “endodontic surgical management” OR “retrograde endodontic treatment” OR “surgical perforation repair” OR “surgical crown root resection.”

Inclusion and Exclusion Criteria

**Figure 1:**
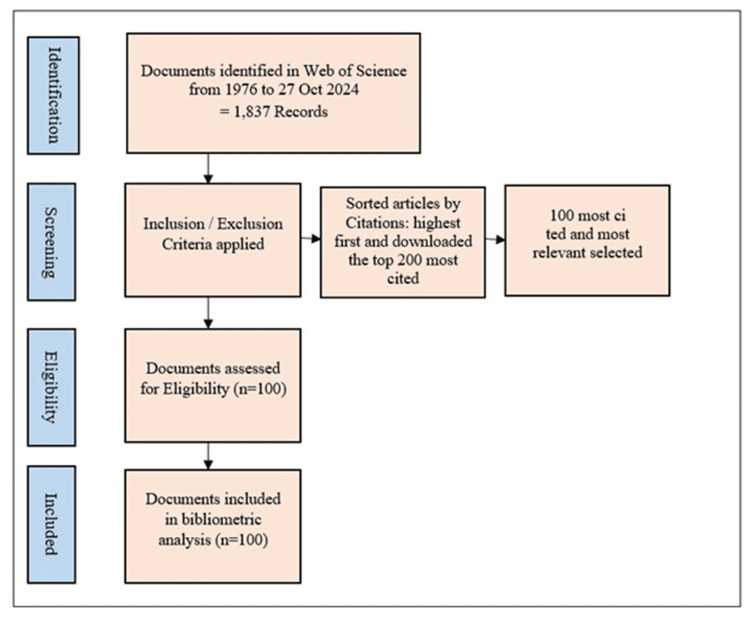
Flowchart illustrating the screening process of articles. WOS: Web of Science database

A WOS search was conducted for publications from 1976 to October 27, 2024. This resulted in 1,837 records. The analysis included only articles and review documents. Documents that were not directly related to ES and meeting abstracts, editorials, letters, and book chapters were excluded. The identified records were sorted by citation count in descending order, and the top 200 most-cited articles were downloaded in the first phase. Two reviewers, AA and SK, independently evaluated the results and selected the top 100 cited articles. Any differences that arose during the screening of the titles were resolved through discussion. The methodology is shown in Figure 1.

Bibliometric Indicators

The 100 most-cited articles were examined, and the following information was extracted: publication year, single or multiple authorship, clinical or non-clinical research, mode of accessibility, LOE, keywords, top-cited authors, institutions, geographic location, and field of study. The study fields included were outcomes of ES, root-end filling materials, bone grafts, membranes and guided tissue regeneration, endodontic microsurgery, imaging, bacterial and histological evaluation, postoperative pain, guided ES intentional replantation, and others. Studies that did not fit in the above fields were grouped under others.

Statistical Analysis

Data analysis was performed using Microsoft Excel version 16 (Microsoft Corp., Redmond, WA, USA). Descriptive statistics, including frequency and percentage, were calculated for all collected variables and displayed as tables and figures. SPSS version 27 (IBM Corp., Armonk, NY, USA) was utilized to evaluate data for single or multiple authorship, clinical or non-clinical research, and mode of accessibility. A p-value less than 0.05 was deemed indicative of statistical significance. Co-author, co-citation, and keyword analyses were conducted using VOS viewer software version 1.6.20.

Ethical Considerations

The King Abdullah International Medical Research Center’s Institutional Review Board authorized the research (reference number: NRR24/062/12). As it was a retrospective examination of open data, ethical approval was not necessary.

Results

Distribution of 100 Most-Cited Articles and Citation Metrics by Year

**Figure 2:**
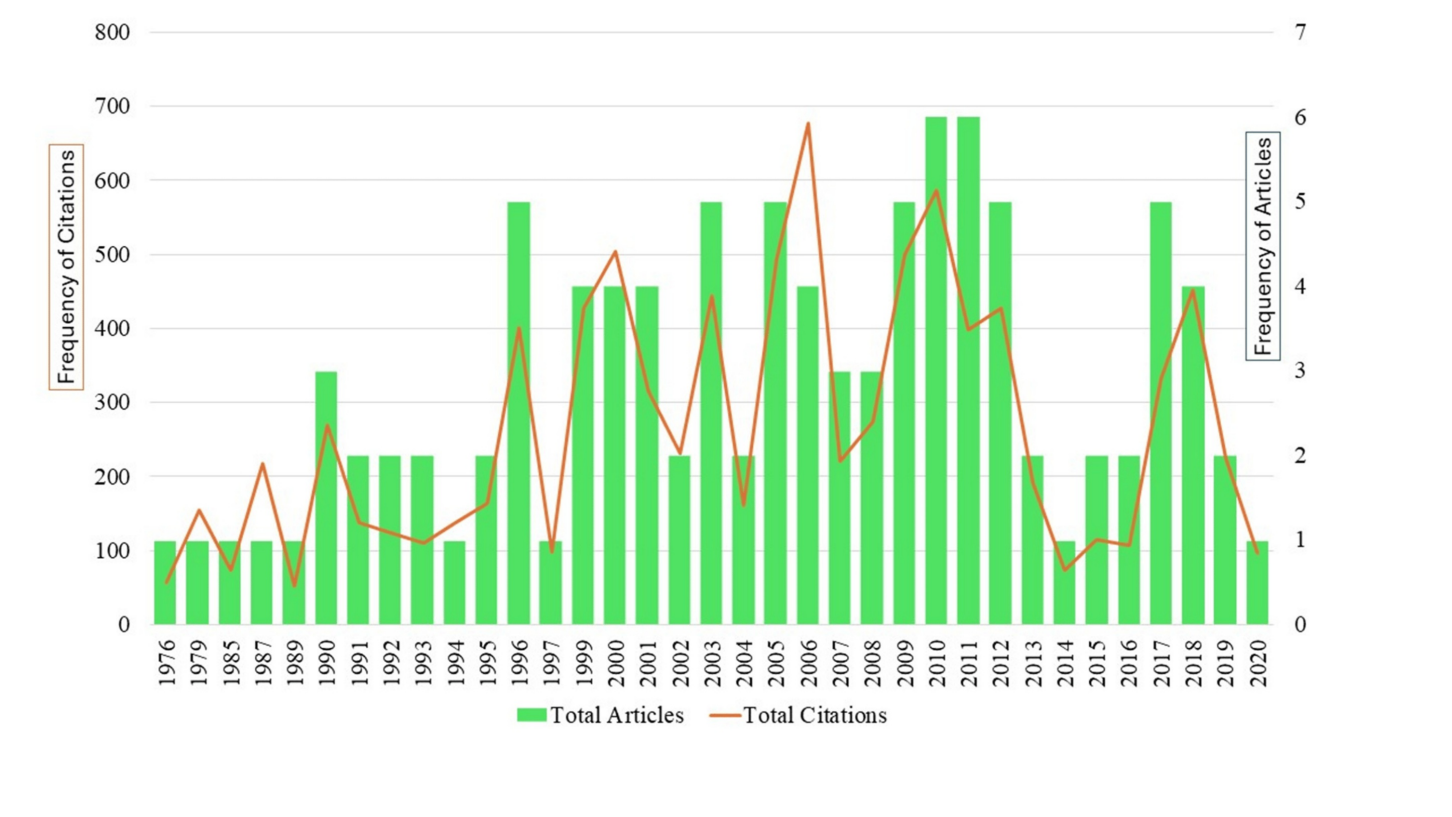
Annual distribution of top-cited publications and citations.

The 100 most-cited publications on ES were published between 1976 and 2020. Not a single article from 2021 made it into the top 100 cited articles. Figure 2 illustrates the annual distribution of publications and citations. For the first 20 years (1976-1995), productivity was low (n = 17), with the first notable increase in 1996, as documented by five studies. A total of 69 papers were published in the following two decades (2001-2020). The most cited articles have received 9,251 citations, averaging 92.51 citations per article. A discernible trend of rising citations is evident, especially post-1990s, culminating in a peak of 677 citations in 2006.

Distribution of Most-Cited Articles Based on Clinical Significance, Accessibility Mode, and Authorship Pattern

The examination of clinical and non-clinical research studies revealed that clinical studies had a markedly greater number of highly cited publications (n = 64) in contrast to non-clinical studies (n = 36). Nonetheless, non-clinical studies exhibited a greater citation impact. The chi-square analysis found no significant statistical correlation between study type (clinical/non-clinical) and citation count (p = 0.13) (Table 1).

**Table 1:** Comparison of the most-cited articles based on clinical significance, accessibility mode, and authorship pattern. P > 0.05: NS, not significant.

Variable	Total articles	Total citations	Citation impact	Chi-square test
Clinical studies	64	5,230	81.72	2.2448, p = 0.13 (NS)
Non-clinical studies	36	4,021	111.69	
Closed access (subscription-based)	91	8,347	91.72	0.0669, p = 0.79 (NS)
Open access	9	904	100.44	
Multiple authors	93	8,637	92.87	0.021, p = 0.88 (NS)
Single authors	7	614	87.71	

Closed-access articles significantly outnumbered open-access ones, with counts of 91 and 9, respectively. On average, citations to open-access papers were somewhat higher than those to closed-access ones. The open-access publications received an average of 100.44 citations each, while the closed-access articles averaged 91.72 citations each. There was no statistically significant difference in articles and citations between closed and open-access publications according to the chi-square analysis (p = 0.79) (Table 1).

In total, 93 articles were authored by multiple authors and garnered 8,637 citations. On average, each publication in this category was cited 93 times. Only seven papers were authored by a single author, yet these works garnered 614 citations, averaging 88 citations per article. Collaborative research articles authored by multiple contributors achieved enhanced citation effect due to their increased visibility within the academic community. The chi-square test revealed no statistically significant relationship between authorship pattern (multiple/single author) and citation count (p = 0.88) (Table 1).

Distribution of the Most-Cited Articles Based on Study Design and LOE

The analysis of publication types reveals that most papers (n = 88) were original research articles, in contrast to review articles (n = 12). Table 2 shows articles distributed by research design and LOE (taken from the modified Oxford Level of Evidence Scale).

**Table 2:** Distribution of articles based on LOE with citation metrics. RCT: randomized controlled trial; LOE: level of evidence

LOE	Study design	Article	Citations	Total articles	Total citations	Citation impact
LOE I	Meta-analysis	7	793	17	1,486	87.41
	RCTs	9	693			
	Systematic review of RCTs	1	54			
LOE II	Clinical trials	7	427	30	2,739	91.30
	Prospective cohort	18	1,751			
	Retrospective cohort	4	345			
	Systematic review	1	216			
LOE III	Case control	2	205	2	205	102.50
LOE IV	Case report	10	791	24	1,953	81.38
	Cross-sectional	14	1,162			
LOE V	Animal study	3	257	27	2,868	106.22
	Laboratory study	12	1,117			
	Review	12	1,494			

According to the LOE, LOE II consisted of over 25% of the articles (n = 29), followed by LOE V (n = 27), LOE IV (n = 24), and LOE I (n = 18). LOE III contained the fewest articles (n = 2). Cohort (22%), cross-sectional (14%), and laboratory studies and reviews (12% each) were the most common research types. Together, meta-analyses (MAs), systematic reviews (SRs), and randomized controlled trials (RCTs) constituted 17% of the research.

LOE II exhibited the maximum number of publications, and cohort studies were chosen as the preferred study design. The most impactful research pertained to LOE V and LOE III, which had high citation impacts of 106.22 and 102.50, respectively.

Author-Wise Distribution of the Most-Cited Articles

In total, 269 authors contributed to the 100 most-cited articles. Of the authors, 85% (n = 229) had written a single paper. Overall, 15 authors contributed to two articles, and 11 authors contributed to three articles. More than three articles were written by 14 authors (Table 3). Most papers were authored by Yonsei University’s Euiseong Kim (n = 11), followed by Syngcuk Kim from the University of Pennsylvania (n = 9) and Minju Song from Yonsei University (n = 7).

**Table 3:** Top authors.

Name of the author and affiliation	Total articles	Total citations	Citation impact
Euiseong Kim - Yonsei University, South Korea	11	729	66.27
Syngcuk Kim - University of Pennsylvania, United States	9	1,455	161.67
Minju Song - Yonsei University, South Korea	7	458	65.43
Thomas von Arx - University of Bern, Switzerland	6	540	90.00
Shimon Friedman - University of Toronto, Canada (4) and Hebrew University of Jerusalem, Israel (2)	6	423	70.50
Massimo Del Fabbro - University of Milan, Italy	5	383	76.60
Silvio Taschieri - University of Milan, Italy	5	383	76.60
Seung-Jong Lee - Yonsei University, South Korea	5	300	60.00
Mahmoud Torabinejad - Loma Linda University, United States	4	644	161.00
Frank C. Setzer - University of Pennsylvania, United States	4	492	123.00
Eyal Rosen - Tel Aviv University, Israel	4	395	98.75
Igor Tsesis - Tel Aviv University, Israel	4	395	98.75
Simon Storgard Jensen - University of Copenhagen, Denmark	4	372	93.00
G. Pecora - University of Pennsylvania, United States	4	316	79.00

The articles authored by Syngcuk Kim from the University of Pennsylvania received the highest citation impact of 161.67, closely followed by Mahmoud Torabinejad from Loma Linda University with a citation impact of 161.00. Shimon Friedman contributed six articles, four under the affiliation of Canada and two under Israel.

“Modern endodontic surgery concepts and practice: a review,” authored by Kim S, gained the highest citations in ES, followed by “Mineral trioxide aggregate and other bioactive endodontic cements: an updated overview - part II: other clinical applications and complications,” authored by Torabinejad M.

Country and Institution-Wise Distribution of the Most-Cited Articles

In total, 12 countries’ authors contributed to more than two articles (Table 4). Eight different countries contributed to one article, while five countries contributed to two articles. Over one-third of articles are from the United States (n = 37). South Korea and Italy rank second and third, with 12 and 10 articles, respectively. The citation impact of England (138.80 citations per article) was the highest, followed by Norway (138.67), the United States (109.14), and Iran (111.25).

**Table 4:** Top countries.

Country	Total articles	Total citations	Citation impact
United States	37	4,038	109.14
South Korea	12	835	69.58
Italy	10	731	73.10
Denmark	9	680	75.56
Switzerland	8	755	94.38
Israel	7	585	83.57
England	5	694	138.80
Sweden	5	492	98.40
Iran	4	445	111.25
Canada	4	285	71.25
Germany	4	274	68.50
Norway	3	416	138.67

A total of 79 institutions were identified. In total, 13 institutes published more than two articles (Table 5). The University of Pennsylvania ranked first with 13 papers, followed by Yonsei University and the University of Milan with 11 and 8 articles, respectively. Loma Linda University (139.20) and the University of Pennsylvania (137.23) exhibited the highest citation impact per article, signifying a substantial influence of their research in the field. This might have resulted from pioneering or highly specialized research.

**Table 5:** Top institutions.

Name of institution	Total articles	Total citations	Citation impact
University of Pennsylvania, United States	13	1,784	137.23
Yonsei University, South Korea	11	729	66.27
University of Milan, Italy	8	589	73.63
University of Bern, Switzerland	6	540	90.00
University of Copenhagen, Denmark	6	487	81.17
Loma Linda University, United States	5	696	139.20
Aarhus University, Denmark	4	253	63.25
Tel Aviv University, Israel	4	395	98.75
United States Department of Defense; United States Air Force	4	277	69.25
University of Toronto, Canada	4	285	71.25
Hebrew University of Jerusalem, Israel	3	190	63.33
IRCCS Istituto Ortopedico Galeazzi, Italy	3	173	57.67
Texas A&M University System, United States	3	282	94.00

Co-Authorship Analysis

**Figure 3:**
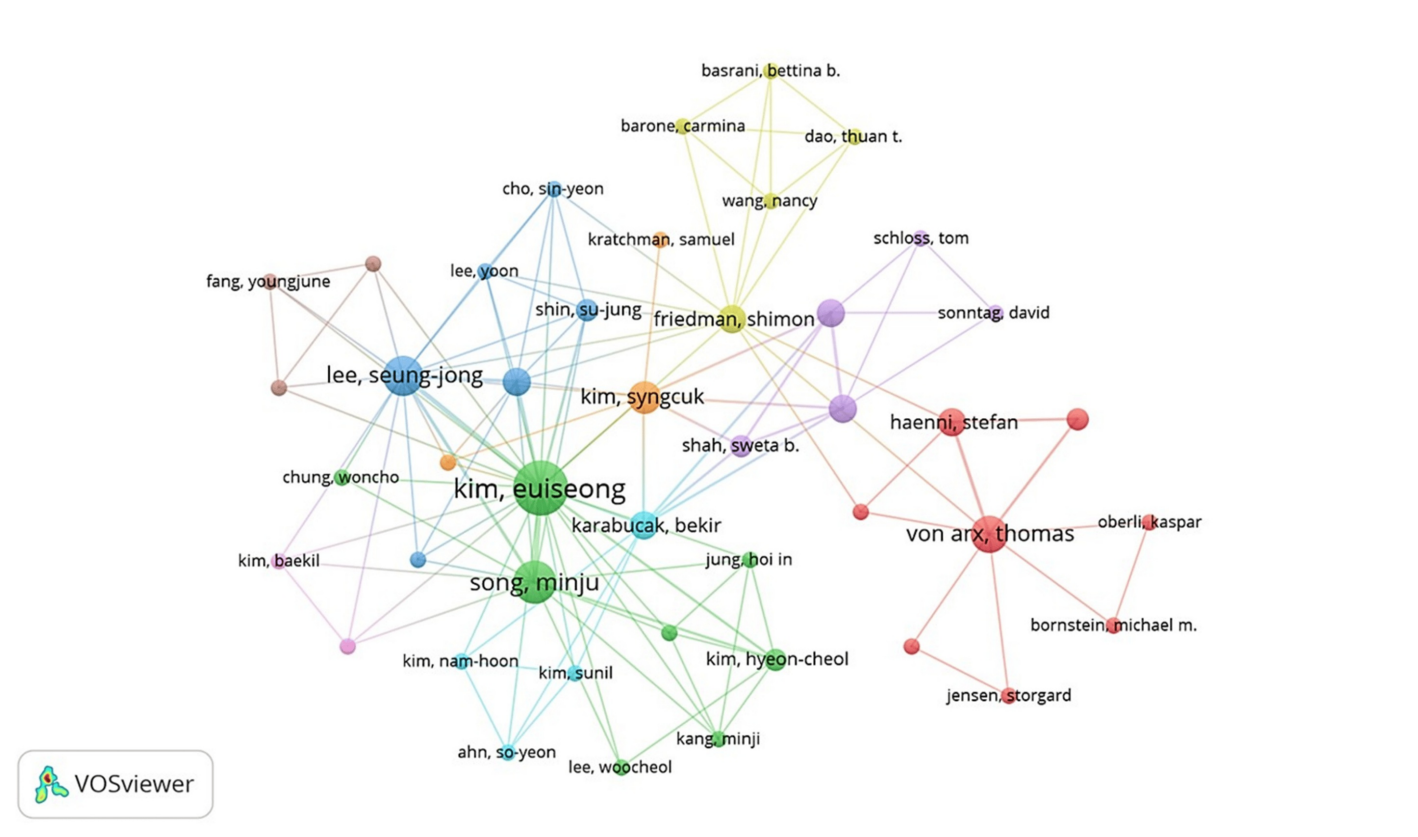
Co-authorship network of publications. The size of the nodes is proportional to the number of publications by the author represented by the node, and the thickness of the edges between nodes reflects the strength of the collaboration between the authors.

In co-authorship networks, nodes represent authors or organizations, which are connected when they share the authorship of a paper. Figure 3 shows the VOSviewer author co-authorship network in the field of ES. The analysis included authors with at least one publication and no minimum requirement for citations. In total, the map highlights 44 authors grouped into nine clusters. Among them, Euiseong Kim from Yonsei University in South Korea stands out as the most collaborative, with 11 papers to his name. His main collaborators include Minju Song (total link strength; TLS = 22), also from Yonsei University, and Hyeon-Cheol Kim (TLS = 8) from Pusan National University. The next most collaborative authors were Seung-jong Lee (TLS = 25) and Minju Song (TLS = 22).

Predominantly Utilized Publishing Sources

The 100 most-cited articles appeared in 14 distinct journal publications, with the majority (n = 95) appearing in the top nine journals, as shown in Table 6. According to the data, the Journal of Endodontics published more than half (n = 54) of the articles. The International Endodontic Journal produced a moderate number of papers (n = 13), yet these articles had a rather high citation impact (107.85).

**Table 6:** Most frequently used journals.

Rank	Name of journal	Total articles	Total citations	Citation impact
1	Journal of Endodontics	54	5,021	92.98
2	International Endodontic Journal	14	1,402	100.14
3	Oral Surgery Oral Medicine Oral Pathology Oral Radiology and Endodontology	13	1,098	84.46
4	Endodontics & Dental Traumatology	4	371	92.75
5	International Journal of Oral and Maxillofacial Surgery	2	307	153.50
6	International Journal of Oral Surgery	2	211	105.50
7	British Dental Journal	2	141	70.50
8	Journal of Oral and Maxillofacial Surgery	2	129	64.50
9	Dentomaxillofacial Radiology	2	126	63.00

**Figure 4:**
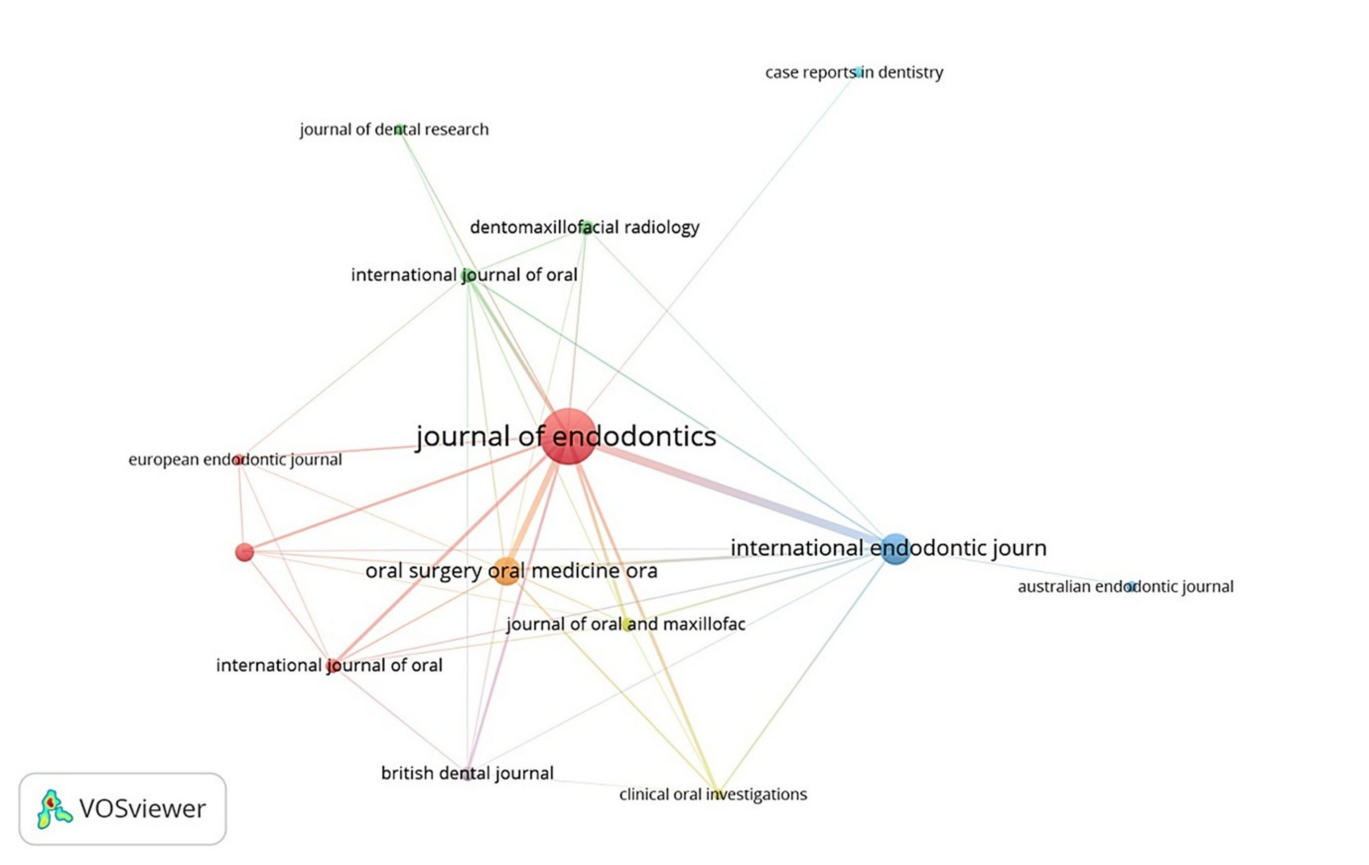
Journal co-citation network of the journals with the highest number of publications on endodontic surgery. The size of the nodes is proportional to the number of times the journal has been cited, and the thickness of the edges is proportional to the strength of the co-citation relationship between the journals.

Figure 4 highlights the VOSviewer co-citation network of the 14 journals that met the citation threshold, forming seven clusters. The Journal of Endodontics topped the list with a TLS of 356, followed by the International Endodontic Journal (TLS = 176) and Oral Surgery Oral Medicine Oral Pathology Oral Radiology and Endodontics Journal (TLS = 142). This highlights local-global collaboration and cross-disciplinary research trends.

Keyword Analysis

The study employed VOSviewer software to analyze the co-occurrence network of keywords. A total of 238 keywords were identified, with 24 keywords meeting the criteria of appearing at least five times in the dataset. Table 7 presents the top 24 keywords along with their frequencies and TLS. The “occurrences” column indicates how often each keyword appears in the articles, while the TLS measures the frequency and intensity of connections between that keyword and others in the dataset.

**Table 7:** Top 24 most co-occurred keywords.

Serial number	Keyword	Occurrences	Total link strength
1	Healing	15	27
2	MTA	14	30
3	Periapical surgery	14	13
4	Endodontic surgery	13	18
5	Outcome	12	34
6	Apical surgery	11	18
7	Endodontic microsurgery	11	27
8	Amalgam	9	18
9	Apicectomy	8	13
10	Apicoectomy	8	22
11	Success	8	25
12	Guided tissue regeneration	7	10
13	Meta-analysis	7	22
14	Root-end filling	7	17
15	success rate	7	19
16	IRM	6	22
17	Microsurgery	6	14
18	Mineral trioxide aggregate	6	15
19	Prognostic factors	6	13
20	Clinical outcome	5	12
21	Follow-up	5	9
22	Periradicular surgery	5	7
23	Retrograde filling	5	6
24	Surgical endodontics	5	7

**Figure 5:**
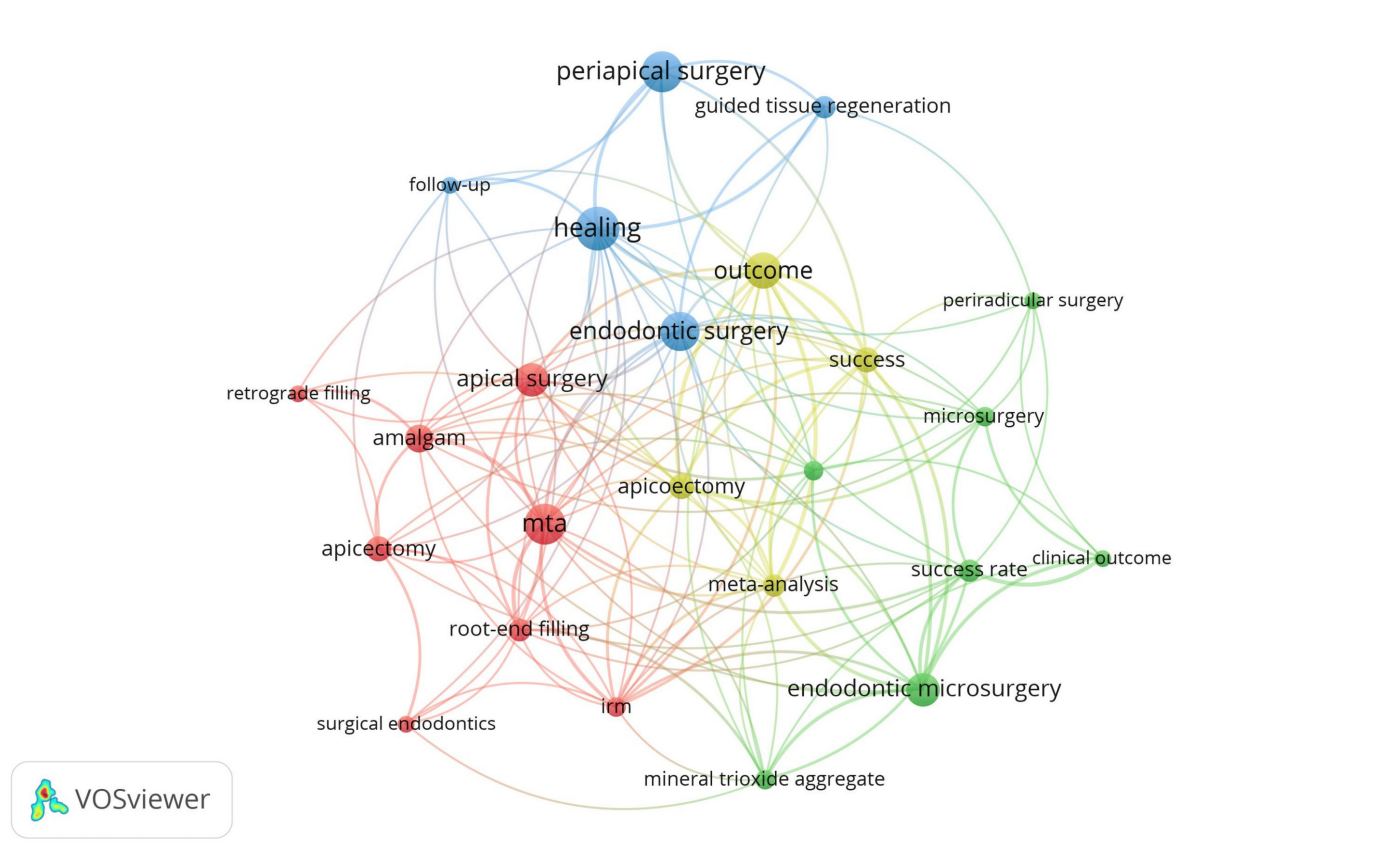
Co-occurrence network of authors’ keywords.

These 24 keywords were grouped into four distinct clusters (Figure 5). Cluster 1 includes eight keywords: amalgam, apical surgery, apicectomy, IRM, MTA, retrograde filling, root-end filling, and surgical endodontics. Cluster 2 contains seven keywords: clinical outcome, endodontic microsurgery, microsurgery, mineral trioxide aggregate, periradicular surgery, prognostic factors, and success rate. Cluster 3 consists of five keywords: endodontic surgery, follow-up, guided tissue regeneration, healing, and periapical surgery. Lastly, Cluster 4 comprises four keywords: apicoectomy, meta-analysis, outcome, and success. These clusters highlight the main research areas within ES, showcasing the evolution of research trends and the prominent themes explored in the most highly cited articles.

Distribution of Most-Cited Articles According to the Field of Study

The most-cited papers were categorized according to their respective fields of study (Table 8). Over 50% of the articles (n = 54) pertained to the field of “outcomes of ES,” followed by “root end filling materials” (n = 23). Articles pertaining to “Endodontic microsurgery” received the highest citation frequency and impact (129.14), followed by “Outcomes of endodontic surgery” (100.39) and “Root end filling materials” (97.61).

**Table 8:** Distribution of articles based on the field of study.

Field of study	Total articles	Total citations	Citation impact
Outcomes of endodontic surgery	31	3,112	100.39
Root end filling materials	23	2,245	97.61
Bone grafts, membranes and guided tissue regeneration	10	805	80.50
Others	8	646	80.75
Endodontic microsurgery	7	904	129.14
Radiography	6	436	72.67
Bacterial and histological evaluation	5	362	72.40
Postoperative pain	4	349	87.25
Guided endodontic surgery	4	285	71.25
Intentional replantation	2	107	53.50

Discussion

This study used a thorough retrospective quantitative bibliometric approach to analyze the bibliometric characteristics of the 100 most-cited publications on ES published in the WoS database during a span of 49 years, from 1976 to 2024. An exciting new age in endodontics, specifically ES, began in the 1990s with the invention of mineral trioxide aggregate (MTA), the first bioceramic material [14]. The increase in publications since the early 1990s suggests that ES research is becoming more and more important. This is probably due to factors such as improvements in magnification, the advancement of biomimetic retro filling materials, the introduction of microsurgical tools, the incorporation of ultrasonics, the expansion of research institutions, better funding opportunities, and more collaborative research projects.

In the present study, articles authored by multiple authors were more frequent and received more citations than single-authored articles. Although not statistically significant, collaborative research articles with multiple authors demonstrated an increased citation impact. The presence of additional authors enhances interest and visibility through their personal networks in the academic community, especially when multiple countries are involved [15].

According to the current research, 36 studies were non-clinical, and 64 studies were clinical. However, non-clinical research had a higher influence on citations. This suggests that non-clinical research had a greater impact on academia, even if clinical research was more prevalent. The findings contradict a study that identified the 100 most-cited publications in regenerative periodontal surgery, with 28 articles classified as clinical and 72 as non-clinical studies [16]. ES has received increased clinical attention as it is structured as scientific trials to learn more about disease, potential therapies, and outcomes.

Our LOE analysis showed that most articles were LOE II (n = 29), followed by LOE V (n = 27) and LOE IV (n = 24). LOE-III contained the fewest articles (n = 2), followed by LOE I (n = 18). Cohort (22%), cross-sectional (14%), and laboratory studies and reviews (12%) were the most common research types. This indicates that the majority of the most-cited articles in ES encompassed study designs of both high and low levels of evidence. Similarly, LOE III and I had the least dispersion of papers, according to bibliometric research by Jamjoom et al. [17] on the LOE analysis of the Saudi Dental Journal’s publications from 2012 to 2021. Rajeh and Khayat [9] highlighted that LOE-I research (MAs, SRs, and RCTs) necessitates advanced skills, adequate resources, considerable time dedication, and professional commitment.

Notably, in the current study, the total citations were the greatest for LOE V papers, especially for review articles. Similar findings were reported in a bibliometric study that examined the 100 most-cited papers in regenerative periodontics surgery [16]. The emergence of innovative bioceramic materials has enabled many laboratory investigations, reviews, and observational studies utilizing them as root-end filling materials, thus establishing this as a significant research domain for scholars in our study.

The Journal of Endodontics published more than half of the articles, followed by the International Endodontic Journal. In an analysis on the most-cited publications in endodontic journals, Fardi et al. [11] observed that the Journal of Endodontics published almost half of the papers. This may be attributed to its status as the oldest journal (1975) and its designation as the official journal of the American Association of Endodontists, rendering it a crucial publication for endodontic specialists.

The United States was the leading country in the top 50 endodontic microbiology classics, according to a study by Karobari et al. [18]. Our research also showed that the United States ranks first in terms of total articles. Despite publishing fewer publications, Norway and England have higher citation impacts. Most of their research designs were funded cohort studies, and they focused on the healing and outcomes after endosurgery and novel root end filling materials.

Euiseong Kim from Yonsei University, South Korea was the most prolific contributor with 11 articles. The publications by Syngcuk Kim from the University of Pennsylvania achieved the highest citation impact, followed by Mahmoud Torabinejad from Loma Linda University. S. Kim is a pioneer in microsurgical endodontics. M. Torabinejad’s remarkable research has resulted in the creation of MTA, which transformed ES. A survey identifying the top 100 endodontic publications in terms of citations designated M. Torabinejad as the most prolific author in the field [19].

The utilization of keywords increases the impact of an article [20]. Keyword mapping can be employed to illustrate the expansion of research in ES. According to the co-occurrence network of keywords analysis, the high frequency keywords in the field of ES includes “Healing,” “MTA,” “Periapical surgery,” “Outcome,” “Endodontic microsurgery,” “Amalgam,” “Apicoectomy,” “Success,” “Guided tissue regeneration” and “root end filling.” Our research is the first of its kind to employ keyword analysis in the context of ES.

The study field with the greatest number of cited articles was outcomes of ES, followed by root-end filling materials. Similarly, Ahmad et al. [21] supported these results, and the majority of the top 50 most-cited publications in the International Endodontic Journal were outcome studies. The emergence of innovative bioceramic materials has enabled many laboratory investigations, reviews, and observational studies utilizing them as root-end filling materials, so establishing this as a significant research domain for scholars in our study.

This study possesses multiple substantial implications. It reveals significant trends and collaborative networks that can guide future research directions. The recognition of important authors, institutions, and nations engaged in this field highlights the significance of global cooperation in progressing endodontic surgery.

Limitations

This analysis was confined to documents obtained from the dataset sourced from WoS. Some significant and relevant articles may have been missed. The study’s reliance on specific keywords for identifying scientific literature may have resulted in the exclusion of relevant research that did not utilize such phrases. Future bibliometric analyses could address these limitations by incorporating additional keywords and databases, offering a broader view of research trends.

Despite these limitations, the findings offer essential guidance for future research in ES. The study highlights influential publications and active institutions, providing a foundation for refining national research strategies.

## Conclusions

This 49-year bibliometric analysis (1976-2024) is the first of its kind that provides an in-depth description of the 100 most influential articles in ES. This research highlights a global dynamic and expanding scholarly landscape, marked by a surge in the last two decades. Articles authored by multiple authors have gathered the highest citations. LOE II exhibited the greatest number of publications. Cohort and cross-sectional studies were the most common research types. The United States and the University of Pennsylvania were the top producers. The Journal of Endodontics published more than half of the articles. Euiseong Kim from Yonsei University in South Korea and Syngcuk Kim from the University of Pennsylvania in the United States were the most prolific authors. Euiseong Kim also stands out as the most collaborative author. The most frequently occurring keywords in the majority of publications were “Healing,” “MTA,” “Periapical surgery,” “Outcome,” “Endodontic microsurgery,” “Amalgam,” “Apicoectomy,” “Success,” “Guided tissue regeneration,” and “root end filling.” These keywords highlighted the most focused areas in the field of ES.

## Appendices

**Table 9:** List of the 100 most-cited articles. EBA: ethoxybenzoic acid; CT: computed tomography; CBCT: cone-beam computed tomography; IRM: intermediate restorative material; MTA: mineral trioxide aggregate; Nd:YAG: neodymium-doped yttrium aluminum garnet

Description of the article	Total citations	Citation density by year (rank)
Modern endodontic surgery concepts and practice: a review [22]	446	23.47 (3)
Mineral trioxide aggregate and other bioactive endodontic cements: an updated overview - part II: other clinical applications and complications [23]	245	35.00 (1)
Observer strategy and the radiographic classification of healing after endodontic surgery [24]	218	5.74 (31)
Outcomes of nonsurgical retreatment and endodontic surgery: a systematic review [25]	216	13.50 (6)
Outcome of endodontic surgery: a meta-analysis of the literature--part 1: comparison of traditional root-end surgery and endodontic microsurgery [26]	206	13.73 (5)
Cytotoxicity of mineral trioxide aggregate using human periodontal ligament fibroblasts [27]	206	8.24 (16)
A prospective clinical study of mineral trioxide aggregate and IRM when used as root-end filling materials in endodontic surgery [28]	185	8.41 (15)
Concentrated growth factors as an ingenious biomaterial in regeneration of bony defects after periapical surgery: a report of two cases [29]	163	27.17 (2)
Long-term follow-up of cases considered healed one year after apical microsurgery [30]	163	7.09 (20)
Periapical surgery [31]	155	3.37 (66)
Prognosis in periradicular surgery: a clinical prospective study [32]	152	6.08 (27)
Outcome of endodontic surgery: a meta-analysis of the literature--part 2:cComparison of endodontic microsurgical techniques with and without the use of higher magnification [33]	141	10.85 (9)
Outcomes of surgical endodontic treatment performed by a modern technique: an updated meta-analysis of the literature [34]	137	11.42 (8)
Apical dentin permeability and microleakage associated with root end resection and retrograde filling [35]	137	4.42 (44)
Short-term observation of the results of endodontic surgery with the use of a surgical operation microscope and Super-EBA as root-end filling material [36]	135	5.19 (37)
Prevalence of persistent pain after endodontic treatment and factors affecting its occurrence in cases with complete radiographic healing [37]	132	6.60 (24)
Persistent periapical radiolucencies of root-filled human teeth, failed endodontic treatments, and periapical scars [38]	125	4.81 (39)
Retrospective evaluation of surgical endodontic treatment: traditional versus modern technique [39]	124	6.53 (25)
Prognostic factors in apical surgery with root-end filling: a meta-analysis [40]	117	7.80 (17)
Incomplete healing (scar tissue) after periapical surgery--radiographic findings 8 to 12 years after treatment [41]	115	3.97 (54)
Periapical bacterial plaque in teeth refractory to endodontic treatment [42]	115	3.29 (68)
Five-year longitudinal assessment of the prognosis of apical microsurgery [43]	114	8.77 (14)
Root end filling materials: a review [44]	114	3.93 (55)
A prospective clinical study of periradicular surgery using mineral trioxide aggregate as a root-end filling [45]	111	6.53 (25)
Prospective clinical study evaluating endodontic microsurgery outcomes for cases with lesions of endodontic origin compared with cases with lesions of combined periodontal-endodontic origin [46]	110	6.47 (26)
Frequency and type of canal isthmuses in first molars detected by endoscopic inspection during periradicular surgery [47]	109	5.45 (34)
Periapical tissue responses and cementum regeneration with amalgam, SuperEBA, and MTA as root-end filling materials [48]	106	5.30 (35)
Analysis of the cause of failure in nonsurgical endodontic treatment by microscopic inspection during endodontic microsurgery [49]	98	7.00 (21)
Bone regeneration with a calcium sulfate barrier [50]	98	3.50 (64)
Endodontic periapical lesion: an overview on the etiology, diagnosis and current treatment modalities [51]	97	19.40 (4)
Considerations in the selection of a root-end filling material [52]	94	3.62 (61)
Detection of the apical lesion and the mandibular canal in conventional radiography and computed tomography [53]	90	3.75 (57)
Sealing ability of amalgam, super EBA cement, and MTA when used as retrograde filling materials [54]	90	3.60 (62)
Long-term results of amalgam versus glass ionomer cement as apical sealant after apicectomy [55]	90	3.00 (72)
Efficacy of low level laser therapy in reducing postoperative pain after endodontic surgery-- a randomized double blind clinical study [56]	89	4.24 (47)
The use of calcium sulphate in the surgical treatment of a 'through and through' periradicular lesion [57]	89	3.71 (58)
Clinical and radiographic assessment of various predictors for healing outcome 1 year after periapical surgery [58]	88	4.89 (38)
Retrograde approaches in endodontic therapy [59]	86	2.53 (77)
Endodontic applications of 3D printing [60]	83	11.86 (7)
Periapical surgery in a Norwegian county hospital: follow-up findings of 477 teeth [61]	83	2.37 (79)
A comparative prospective randomized clinical study of MTA and IRM as root-end filling materials in single-rooted teeth in endodontic surgery [62]	81	4.05 (53)
Comparison of clinical outcome of periapical surgery in endodontic and oral surgery units of a teaching dental hospital: a retrospective study [63]	81	3.38 (65)
Prognostic factors for clinical outcomes in endodontic microsurgery: a retrospective study [64]	80	5.71 (32)
Vestibular surgical access to the palatine root of the superior first molar: “low-dose cone-beam” CT analysis of the pathway and its anatomic variations [65]	80	3.64 (60)
Guided modern endodontic surgery: a novel approach for guided osteotomy and root resection [66]	78	9.75 (12)
Guided tissue regeneration in periapical surgery [67]	78	5.20 (36)
Outcome of surgical endodontic treatment performed by a modern technique: a meta-analysis of literature [68]	76	4.75 (40)
Comparison of long-term survival of implants and endodontically treated teeth [69]	74	6.73 (23)
The guided tissue regeneration principle in endodontic surgery: one-year postoperative results of large periapical lesions [70]	74	2.47 (78)
Efficacy of xenogeneic bone grafting with guided tissue regeneration in the management of bone defects after surgical endodontics [71]	73	4.06 (52)
Success and failure in periradicular surgery: a longitudinal retrospective analysis [72]	73	2.81 (74)
Posterior endodontic surgery: anatomical considerations and clinical techniques [73]	73	1.83 (87)
Randomized clinical trial of root-end resection followed by root-end filling with mineral trioxide aggregate or smoothing of the orthograde gutta-percha root filling--1-year follow-up [74]	72	4.50 (43)
A bacteriological and histological evaluation of 58 periapical lesions [75]	72	2.18 (84)
Chronic facial pain associated with endodontic therapy [76]	72	2.06 (85)
A comparison of 2- and 3-dimensional healing assessment after endodontic surgery using cone-beam computed tomographic volumes or periapical radiographs [77]	71	8.88 (13)
Comparison of mineral trioxide aggregate and iRoot BP Plus root repair material as root-end filling materials in endodontic microsurgery: a prospective randomized controlled study [78]	71	8.88 (13)
Attachment and morphological behavior of human periodontal ligament fibroblasts to mineral trioxide aggregate: a scanning electron microscope study [79]	71	3.38 (65)
Targeted endodontic microsurgery: a novel approach to anatomically challenging scenarios using 3-dimensional-printed guides and trephine burs-a report of 3 cases [80]	70	10.00 (11)
Evaluation of pathologists (histopathology) and radiologists (cone beam computed tomography) differentiating radicular cysts from granulomas [81]	69	4.60 (41)
Molecular analysis of persistent periradicular lesions and root ends reveals a diverse microbial profile [82]	69	4.31 (45)
Leakage evaluation of root end filling materials using endotoxin [83]	69	3.00 (72)
Periapical radiography and cone beam computed tomography for assessment of the periapical bone defect 1 week and 12 months after root-end resection [84]	67	4.19 (49)
Periapical endodontic surgery: a 3-year follow-up study [85]	67	3.05 (71)
Periapical resurgery versus periapical surgery: a 5-year longitudinal comparison [86]	66	3.30 (67)
Clinical management of nonhealing periradicular pathosis. Surgery versus endodontic retreatment [87]	66	2.28 (82)
Treatment outcome in endodontics: the Toronto study--phases 3, 4, and 5: apical surgery [88]	64	4.27 (46)
Endodontic microsurgery using dynamic navigation system: a case report [89]	62	10.33 (10)
Periapical and periodontal healing after osseous grafting and guided tissue regeneration treatment of apicomarginal defects in periradicular surgery: results after 12 months [90]	61	2.77 (75)
Diagnostic validity of periapical radiography and CBCT for assessing periapical lesions that persist after endodontic surgery [91]	59	7.38 (19)
Long-term outcome of the cases classified as successes based on short-term follow-up in endodontic microsurgery [92]	59	4.54 (42)
Effect of platelet concentrate on quality of life after periradicular surgery: a randomized clinical study [93]	59	4.54 (42)
Periapical surgery and the maxillary sinus: radiographic parameters for clinical outcome [94]	59	3.28 (69)
Outcome of nonsurgical retreatment and endodontic microsurgery: a meta-analysis [95]	58	5.80 (30)
Effect of guided tissue regeneration on the outcome of surgical endodontic treatment: a systematic review and meta-analysis [96]	58	4.14 (50)
Clinical outcome of endodontic microsurgery that uses EndoSequence BC root repair material as the root-end filling material [97]	57	5.70 (33)
Comparative evaluation of platelet-rich plasma and guided tissue regeneration membrane in the healing of apicomarginal defects: a clinical study [98]	57	4.07 (51)
Endodontic surgery using 2 different magnification devices: preliminary results of a randomized controlled study [99]	56	2.95 (73)
Postoperative discomfort associated with surgical and nonsurgical endodontic retreatment [100]	56	2.24 (83)
Follow-up study of apicoectomized molars [101]	56	1.14 (92)
A prospective randomized controlled study of mineral trioxide aggregate and super ethoxy-benzoic acid as root-end filling materials in endodontic microsurgery [102]	55	4.23 (48)
Periapical healing of mandibular molars after root-end sealing with dentine-bonded composite [103]	55	2.29 (81)
Use of dental operating microscope in endodontic surgery [104]	55	1.72 (88)
Periodontal tissue regeneration including cementogenesis adjacent to dentin-bonded retrograde composite fillings in humans [105]	55	1.72 (88)
Computer-aided design/computer-aided manufacturing-guided endodontic surgery: guided osteotomy and apex localization in a mandibular molar with a thick buccal bone plate [106]	54	7.71 (18)
Retention and healing outcomes after intentional replantation [107]	54	6.00 (28)
Endodontics, endodontic retreatment, and apical surgery versus tooth extraction and implant placement: a systematic review [108]	54	6.75 (22)
Prognostic factors of clinical outcomes in endodontic microsurgery: a prospective study [109]	54	4.50 (43)
Outcomes of endodontic micro-resurgery: a prospective clinical study [110]	54	3.86 (56)
Survival rate of teeth with a C-shaped canal after intentional replantation: a study of 41 cases for up to 11 years [111]	53	5.89 (29)
Endodontic applications of guided tissue regeneration in endodontic surgery [112]	54	1.86 (86)
Clinical results with two different methods of root-end preparation and filling in apical surgery: mineral trioxide aggregate and adhesive resin composite [113]	53	3.53 (63)
Biocompatibility of retrograde root filling materials: a review [114]	53	3.12 (70)
Healing of apical periodontitis in dogs after apicoectomy and retrofilling with various filling materials [115]	53	1.83 (87)
Effects of Nd:YAG laser on apical seal of teeth after apicoectomy and retrofill [116]	52	1.58 (89)
Treatment results of apical surgery in premolar and molar teeth [117]	52	1.53 (90)
Leakage in vitro with IRM, high copper amalgam, and EBA cement as retrofilling materials [118]	52	1.44 (91)
Persistent extraradicular infection in root-filled asymptomatic human tooth: scanning electron microscopic analysis and microbial investigation after apical microsurgery [119]	51	3.64 (59)
A comparative study of the biocompatibility of three root-end filling materials in rat connective tissue [120]	51	2.68 (76)
Molar apicectomy with amalgam root-end filling: results of a prospective study in two district general hospitals [121]	51	2.32 (80)
